# CircASPH Promotes Hepatocellular Carcinoma Progression Through Methylation and Expression of HAO2

**DOI:** 10.3389/fonc.2022.911715

**Published:** 2022-06-20

**Authors:** Han Zhuo, Jinguo Xia, Jin Zhang, Junwei Tang, Sheng Han, Qitong Zheng, Deming Zhu, Feihong Zhang, Zhenggang Xu, Dongwei Sun, Zhongming Tan, Chen Wu

**Affiliations:** ^1^ Hepatobiliary Center, The First Affiliated Hospital of Nanjing Medical University, Nanjing, China; ^2^ School of Medicine, Jiangsu University, Zhenjiang, China; ^3^ General Surgery, The First Affiliated Hospital of Nanjing Medical University, Nanjing, China

**Keywords:** HCC, circASPH, miR-370-3p, DNMT3b, methylation

## Abstract

CircRNAs have been reported to be related to hepatocellular carcinoma (HCC) development. Limited studies have revealed the expression profile of circRNAs in tumor and para-tumor normal samples in HCC patients. We found that circASPH was significantly increased in HCC tumor samples and that the level of circASPH was closely related to the overall survival of HCC patients. Mechanistically, circASPH could regulate the methylation of the promoter and expression of hydrocyanic oxidase 2 (HAO2) to promote HCC progression by acting as a sponge for miR-370-3p, and miR-370-3p could target DNMT3b and increase the 5mC level. In summary, our study determined that circASPH could regulate the methylation and expression of HAO2 and it could be considered an important epigenetic regulator in HCC progression.

## Introduction

Hepatocellular carcinoma (HCC) is becoming one of the most common malignancies, with a high rate of cancer-related death worldwide, increasing the burden worldwide (1). Hepatitis C virus (HCV) and hepatitis B virus (HBV) infections and non-alcoholic fatty liver are risk factors that result in HCC (2–4), as well as excessive drinking, genetic factors, smoking, excess body weight, and type 2 diabetes (5–7). HCC possesses its unique characteristics because the prognosis depends on the tumor stage and severity of the liver disease. The interventions include liver transplantation and surgical resection, which are only effective in the early stages (8). Therefore, the mechanism of HCC development should be uncovered to improve patient prognosis and treatment effects.

Circular RNAs (circRNAs) have been identified as a new type of non-coding RNAs. CircRNAs have a covalently closed loop structure without a 5’ cap and a 3’ tail, which is different from traditional linear RNA (9). Because of their special structure, circRNAs could be resistant to ribonuclease cleavage, making them more stable (10–12). At the same time, circRNAs have developmental stage–related patterns, tissue specificity, and a high species conservation (13–15). Thereby, circRNAs have been used as biomarkers in disease diagnosis, prognosis evaluation, and progress monitoring. CircRNAs are divided into exon circular RNAs (EcircRNAs), exon–intron circular RNAs (EIciRNAs), and intron circular RNAs (ciRNAs), depending on sequences spliced from mRNA (9). More and more circRNAs are identified and verified to play a crucial role in cancers, especially in HCC. For instance, hepatocyte nuclear factor 4 alpha can activate circ_104075 transcription by binding to its promoter, sponging miR-582-3p to upregulate Yes-associated protein (YAP) to advance the process of HCC (16). CircTMEM45A could interact with miR-665 to inhibit the expression of insulin-like growth factor 2 to promote HCC tumorigenesis (17). However, the role of circASPH remains unclear in HCC.

DNA methylation is catalyzed by two types of enzymes. In the first type, after DNA replication, DNA methyltransferase 1 (DNMT1) and its cofactor could recognize hemimethylated DNA (18). In the second type, DNMT3A and DNMT3B, together with their coactivator DNMT3L, catalyze new methyl groups to unmethylated cytosines (19). However, the mechanism of the DNMT3b/5mC axis in the regulation of HCC remains unresolved. HAO2, hydroxy acid oxidase 2, is a member of the flavoenzyme family that is responsible for the oxidation of l-2-hydroxy acids to ketoacids at the expense of molecular oxygen. HAO2 is downregulated in HCC and can be used to predict metastasis and poor survival (20). HAO2 is demonstrated to inhibit the malignancy of clear cell renal cell carcinoma (21). The methylation of HAO2 in HCC remains unclear.

Here, two circRNA-seq databases were used to identify whether circASPH was significantly upregulated in tumor tissues in HCC patients. The expression of circASPH levels in tumor tissues was closely related to the prognosis of HCC patients. Mechanistically, circASPH could regulate the methylation process and expression of HAO2 by acting as a sponge for miR-370-3p, and miR-370-3p targeted DNMT3b to increase the 5mC level. Therefore, circASPH could be considered an important epigenetic regulator in HCC progression.

## Materials and Methods

### Clinical Data

In total, 181 samples were collected from consecutive patients with HCC who underwent curative resection in the Hepatobiliary Center, The First Affiliated Hospital of Nanjing Medical University. Fresh human HCC and adjacent nontumor liver tissue samples were blindly collected from the cohort. Informed consent was obtained from each patient, and ethical approval was granted by the Ethics Committee of The First Affiliated Hospital of Nanjing Medical University. The information is summarized in **Table 1**.

**Table 1:** Clinicopathological features of HCC patients (n = 20).

Patients	n (%)
**Age (years)**	
<60	12 (60.0)
≥60	8 (40.0)
**Gender**	
Male	14 (70.0)
Female	6 (30.0)
**T stage**	
T1–T2	9 (45.0)
T3–T4	11 (55.0)
**Regional lymph node metastasis**	
Yes	16 (80.0)
No	4 (20.0)
**Distance metastasis**	
Yes	3 (15.0)
No	17 (85.0)
**Tumor size**	
< 5 cm	11 (55.0)
≥ 5 cm	9 (45.0)

### Cell Culture, RNA Extraction, and Transfection

The human HCC cell lines Huh-7, SMMC-7721, HepG2, MHCC97H, and HCCLM3 were routinely maintained in our laboratory. The normal liver cell line was THLE-2. Transfection was carried out by using Lipofectamine 3000 (Invitrogen, Invitrogen, Carlsbad City, CA, USA), and total RNA was obtained according to the manufacturer’s instructions. Si-circASPH (5’-GCAAAAGGACUUUAAAGAGAUU-3’) and miR-370-3p mimics were purchased from GenePharma (GenePharma, Shanghai, China).

### Plasmid Construction

The lentiviral vectors pGMLV-SC5-shmiR-370-3p and pGMLV-SC5-shRNA-circASPH and negative sequences were purchased from GenePharma (China). The lentiviral cDNA templates of circASPH and DNMT3b were cloned into the pPB-CAG vector according to the manufacturer’s instructions. Cells were then transduced with the appropriate lentivirus.

### RT-qPCR

MiRNAs were extracted with the mirVana™ miRNA Isolation Kit (Life Technologies, Carlsbad City, CA, USA) (Genewiz, Shanghai, China). Real-time PCR was performed in triplicate with the SYBR Green PCR method. All miRNA levels were normalized to the U6 small nuclear RNA level; other RNA levels were adjusted using 18S as the reference. Relative expression was analyzed by the comparative cycle threshold (Ct) method. Primer sequences are listed below (Genewiz, China): circASPH, forward: 5’- ACTGCTCCCCCTGAGGAT-3, reverse: 5’- GGGACTGCTGGCTCTGAA-3’; miR-370-3p, forward: 5’-AGACCCCGCTATGGCTCTATT-3’, reverse: 5’- TTTTGGCATAACTAAGGCCGAA -3’; U6, forward: 5’- TGTAACCAGAGAGCGGGATGT-3’, reverse: 5’-AACGCTTCACGAATTTGCGT-3’. 18SrRNA, forward: 5’-CAGCCACCCGAGATTGAGCA-3’, reverse: 5’-TAGTAGCGACGGGCGGTGTG-3’; DNMT3b, forward: 5’-AGGGAAGACTCGATCCTCGTC-3’, reverse: 5’- GTGTGTAGCTTAGCAGACTGG-3’; HAO2, forward: 5’-GCATCACGCGGGATGACAA-3’, reverse: 5’- GCGATACAAATAGGGGCACTGA-3’.

### Western Blotting Analysis

The protein was extracted by using Radio-Immunoprecipitation Assay (RIPA) lysis buffer. After being centrifuged, the concentration of protein was checked by a BCA kit. Proteins were separated by Sodium Dodecyl Sulfate Polyacrylamide Gel electropheresis (SDS-PAGE) and transferred to Polyvinylidenefluoride (PVDF) membranes (Millipore, Boston, MA, USA). DNMT3b (#57868, CST, Danvers, MA, USA), HAO2 (ab229817, abcam, UK), and β-actin (#3700, CST, USA) antibodies were incubated overnight. After the incubation of the secondary antibodies, the bands were used in Image Lab after adding a chemiluminescent substrate (Millipore, USA) to visualize. Image Lab software was used to analyze the results.

### Immunofluorescence and Immunocytochemistry Assay

For 5mC (ab10805, abcam, United Kingdom) and 5hmC (ab106918, abcam, United Kingdom) staining, the treated cells or tissues were fixed with 4% PFA and washed with PBS, then permeabilized with 0.3% Triton X-100 (Beyotime, China). For immunofluorescence, after blocking, the cells or tissues were incubated with the primary antibody (Abcam, UK) overnight at 4°C. Cells or tissues were incubated with secondary antibodies and DAPI. Images were taken under the microscope. For immunocytochemistry, 3% hydrogen peroxide was used to block the endogenous peroxidase. After being washed with PBS, the tissues were incubated with 10% goat serum and the primary antibody was added for 4°C overnight. After incubating with the secondary antibody, a DAB solution was added for color staining.

### Luciferase Assay

The wild-type (WT) and mutant (MUT) 3′-UTR fragment sequences of circASPH and human DNMT3b were constructed into pmirGLO vectors (Genewiz, China). Each cell was seeded with a proper density in 12-well plates. Alternatively, the cells were co-transfected with plasmids and mimics. After transfection, the cells were harvested and lysed. Luciferase activity was recorded using the dual-luciferase reporter assay kit (Promega, USA).

### FISH Assay

A FISH kit was used for the FISH assay. Specific probes for circASPH and miR-370-3p were synthesized by GenePharma (China). Briefly, cells were fixed with 4% paraformaldehyde, treated with 0.5% Triton X-100 (Beyotime, Shanghai, China), and incubated with circASPH and miR-370-3p probes overnight. Then, cell nuclei were stained with DAPI (Yeasen, Shanghai, China). Images were obtained with a fluorescence microscope. The sequences of probes were: circASPH probe 5′-TCTCTCTTTAAGTCCTTTTGCTTTTTGTTC-3′ and miR-370 probe 5′-ACCAGGTTCCACCCCAGCAGGC-3′.

### CCK8 Assay

Transfected BC cells at a density of 2,000 were seeded in 96-well plates. A CCK8 assay kit (Sigma, Carlsbad City, CA, USA) was added and incubated with cells. Then, the absorbance was measured by a microplate reader at five time periods.

### Wound-Healing Assay

Transfected HCC cells were seeded in six-well plates. A 200 μl pipette tip was used to generate a linear gap. After 24 h, we used the microscope to take pictures and measured the width (W) of the scratch wound. The rate of close distance of the wounds was calculated. All measurements were carried out three times.

### Transwell Assay

The cell suspension was added into Transwell chamber inserts (Millipore, USA) with Matrigel. Approximately 24 h later, cells were stained and pictures taken were used to measure invasion assays under a microscope.

### Biotin-Coupled RNA Capture

SMMC-7721 cells were transfected with biotinylated circASPH or miRNA-370-3p (GenePharma, China) according to the standard protocol of Lipofectamine™ 3000 (Invitrogen, Carlsbad City, CA, USA). After transfection, cells were harvested and lysed. Streptavidin-conjugated magnetic beads were activated and blocked with a blocking buffer for 2 h. Then, the beads were incubated with cell lysates at 4°C for 8 h to pull down the biotin-coupled RNA complex. The level of RNA was evaluated by PCR.

### MeDIP Assay

Methylation was detected by the MeDIP kit (Abcam, London, United Kingdom) by applying a non-cross-reactive 5mC antibody. In the assay of the kit, DNA is sheared and then added into a microplate well and an antibody specific to methylcytosine is then used to capture methylated DNA in the wells. Then, the specificity of enriched methylated DNA was evaluated by quantitative PCR.

### Migration and Invasion Assays and *In Vivo* Metastasis Assays

The HCC cell lines were transfected with shRNA-circASPH and negative control (NC). Next, HCC cell lines were collected for subcutaneous injection. BALB/c nude mice were randomly divided into two groups. Mice in the control groups received subcutaneous injection with control cells, and experimental groups received a subcutaneous administration of treated HCC cells. After 5 weeks, the imaging of mice was performed and the tumor volume and weight were examined. The tumor volume was measured and calculated as follows: V = (length × width^2^)/2. All animal experiments were approved by the Committee on the Ethics of Animal Experiments of Nanjing Medical University.

### Target Prediction and Bioinformatics

The TargetScan (http://www.targetscan.org), miRBase (http://www.mirbase.org), and miRanda (www.microrna.org) algorithms were used to screen for the targets of miRNAs. Circbase (http://circbase.org/) was used to predict the interaction between circRNAs and miRNAs. Previously published RNA-Seq and BS-Seq datasets (GSE55752) were re-analyzed. RNA-Seq reads were trimmed using Trim Galore and mapped to the GRCh38 human genome using HISAT2. Read count quantification was performed with HTSeq-count. Differential gene expression analysis was conducted with DESeq2. BS-Seq data were mapped with Bismark, and differential methylation regions were identified by Diffbind. The distribution of DMRs was analyzed by ChiPseeker and deepTools.

### Statistical Analysis

Statistical analysis was performed with SPSS 19.0 software (SPSS). All tests were two tailed, and p<0.05 was considered statistically significant.

## Results

### CircASPH Expression Is Significantly Upregulated in HCC Tissues and Related to Patient Prognosis

**Figure 1:**
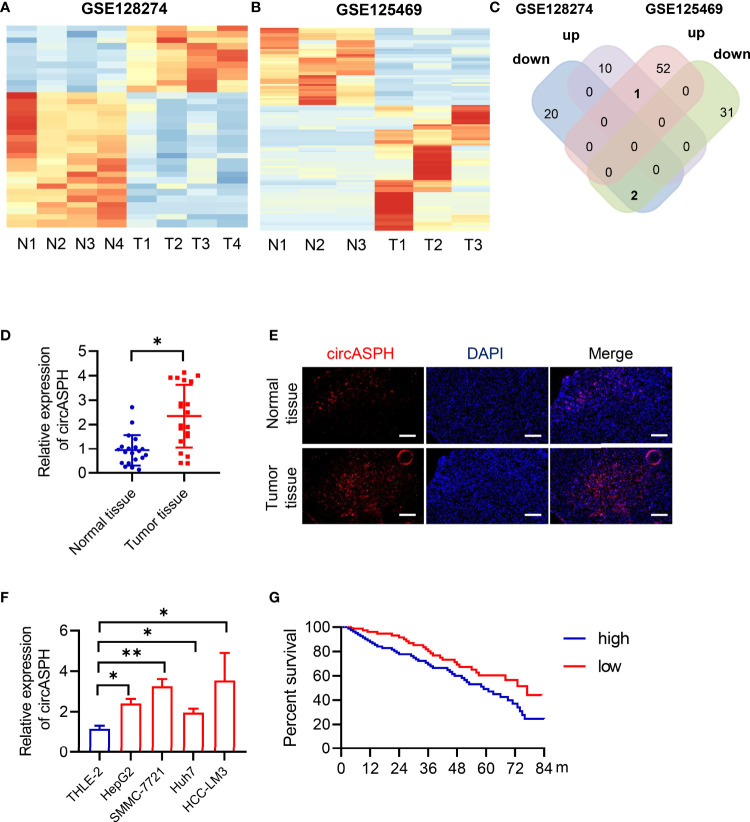
CircASPH expression was identified to be significantly upregulated in HCC tissues and related to patient prognosis. **(A, B)** Heat maps of differentially expressed circRNAs in HCC tissues and non-tumor tissues obtained from GSE128274 and GSE125469. **(C)** The numbers of overlapping differentially expressed circRNAs from two GEO databases are shown in the Venn diagram. **(D)** The level of circASPH expression was evaluated by RT-qPCR in 20 paired HCC tissues and normal tissues. **(E)** FISH analysis of the expression of circASPH in HCC tissues and normal tissues. **(F)** CircASPH expression in HCC cell lines compared with THLE-2 cells. **(G)** Kaplan–Meier analysis showed that the level of circASPH was predictive of overall survival. *p < 0.05, **p < 0.01.

To identify the differentially expressed circRNAs between normal tissues and HCC tumor tissues, we used two GEO databases (GSE28274 and GSE125469) for investigation (**Figures 1A, B**). The circRNAs with significant differential expression (fold change ≥2.0 and P < 0.05) between the groups were identified. From the results of two GEO databases, one circRNA was preliminarily identified to be upregulated, while two circRNAs were downregulated in the HCC tissue samples compared to the para-tumor normal samples (**Figure 1C**). In this study, we chose circASPH for further investigation.

To determine whether circASPH was associated with HCC, firstly, RT-qPCR was used to indicate that the expression of circASPH was higher in HCC tissues (**Figure 1D**). Then, we used the FISH assay to identify the expression of circASPH in HCC tumor tissues. It showed that the level of circASPH expression was higher in tumor tissues (**Figure 1E**). We also investigated the level of circASPH in several HCC cell lines. The level of circASPH was higher in HCC cell lines (HepG2, SMMC-7721, Huh7, and HCCLM3) (**Figure 1F**). Then, we analyzed the relationship between the circASPH level and HCC patient prognosis. Multivariate Cox analysis revealed that the circASPH level in HCC tissue was an independent prognostic factor in HCC patients (**Figure 1G**). These data suggest that circASPH plays an important role in HCC tumorigenesis and progression.

### CircASPH Promotes Cell Proliferation, Migration, and Invasion in HCC Cells

**Figure 2:**
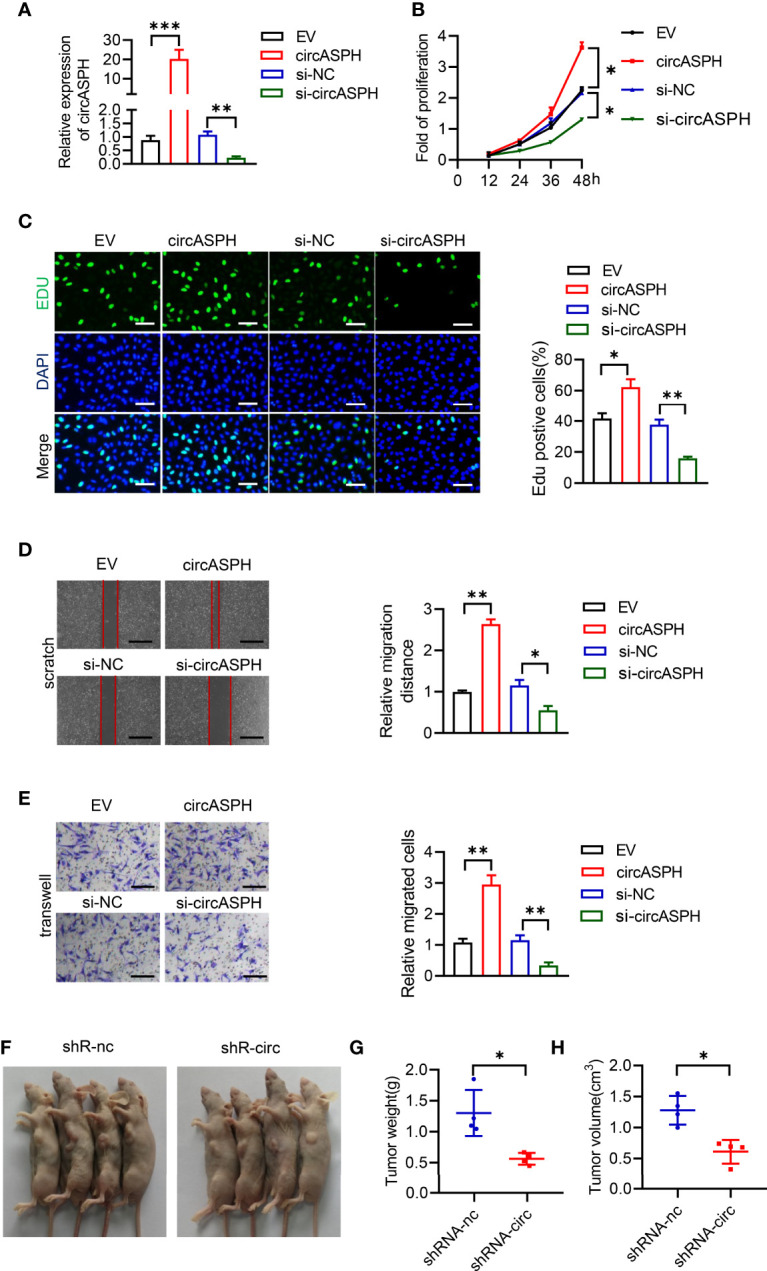
CircASPH promoted HCC cell proliferation, migration, and invasion. **(A)** The expression of the circASPH level was detected by circASPH overexpressed and knocked down in HCC cells. **(B, C)** Cell Counting Kit-8 (CCK-8) assays and EDU staining assays were used to indicate the function of circASPH in the proliferation of HCC cells. **(D)** Wound-healing assays showed the role of circASPH in the migration of HCC cells. **(E)** Transwell assays showed the function of circASPH in the invasion of HCC cells. **(F–H)** Xenograft tumors composed of shRNA-circASPH-transfected HCC cells were significantly smaller than those composed of shRNA-NC-transfected HCC cells. *p < 0.05, **p < 0.01, ***p < 0.001.

To investigate the biological function of circASPH in HCC progression, circASPH was stably overexpressed and knocked down in SMMC-7721 cell lines (**Figure 2A**). The CCK8 assay and EDU staining assay were carried out to verify the proliferation of HCC cells. The results indicated that the cell proliferation was enhanced by circASPH overexpression, whereas it was reduced by circASPH knockdown (**Figures 2B, C**). Meanwhile, wound-healing assays were performed to evaluate the cell migration ability and Transwell assays were used to examine the cell invasion. The results showed that the migration and invasion capacities were significantly increased after circASPH overexpression. However, the ability of cell migration and invasion capacities were reduced when the circASPH level was decreased *in vitro* (**Figures 2D, E**). To further determine whether circASPH induced the tumor growth *in vivo*, we injected control or shRNA-circASPH-transfected cells into nude mice. Our results revealed that the tumors composed of shRNA-circASPH-transfected cells were significantly smaller than those in control cells (**Figures 2F–H**). Therefore, we verified that circASPH functioned as a tumor promoter to enhance the proliferation, invasion, and metastasis of HCC *in vitro* and *in vivo*.

### CircASPH Regulates the Level of the DNMT3b/5mC Axis by Sponging miR-370-3p in HCC Cells

**Figure 3:**
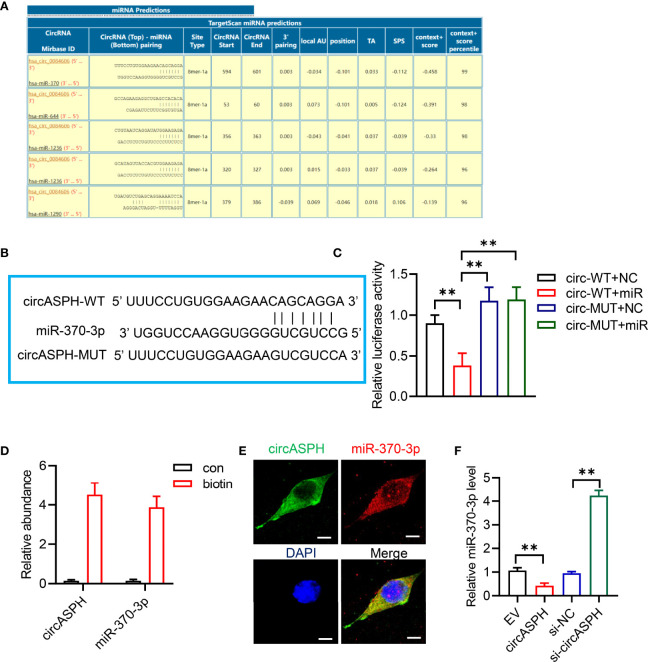
CircASPH sponged miR-370-3p. **(A)** Score list for the circInteractome of predicting miRNAs sponged by circASPH. **(B)** Predicted binding sites between circASPH and miR-370-3p. **(C)** Dual-luciferase reporter assay showed that the co-transfection of WT and mimic miR-370-3p markedly decreased the luciferase activity. **(D)** MiR-370-3p was abundantly pulled down by a circASPH probe. **(E, F)** Images of circASPH and miR-370-3p co-localized in the cytoplasm; scale bar, 20 μm. *p < 0.05, **p < 0.01, ***p < 0.001.

CircRNAs have been reported to function as sponges for miRNAs. Therefore, we investigated the role of circASPH to function as a sponge for miRNAs. Candidate targets were determined using target prediction. According to the predicted results, miR-370-3p gained the highest score (**Figure 3A**). Then, we used molecular biological methods to investigate the direct interaction between circASPH and miR-370-3p. The 3′-UTR fragment of circASPH containing the putative binding site [wild type (WT)] of miR-370-3p or a mutant sequence was cloned into pmirGlo vectors. The results showed that, when co-transfected with WT and NC or mimic, the mimic miR-370-3p significantly decreased the luciferase activity (**Figures 3B, C**). We also designed a linear biotinylated circASPH probe. Our RT-qPCR results showed that miR-370-3p was abundantly pulled down by the circASPH probe in HCC cells (**Figure 3D**). We also used the FISH assay to detect the interaction between the circASPH and miR-370-3p. The result showed that circASPH and miR-370-3p could be co-localized (**Figure 3E**). At the same time, overexpressing circASPH could decrease the miR-370-3p expression level, while the reduction of miR-370-3p was induced by circASPH knockdown (**Figure 3F**).

**Figure 4:**
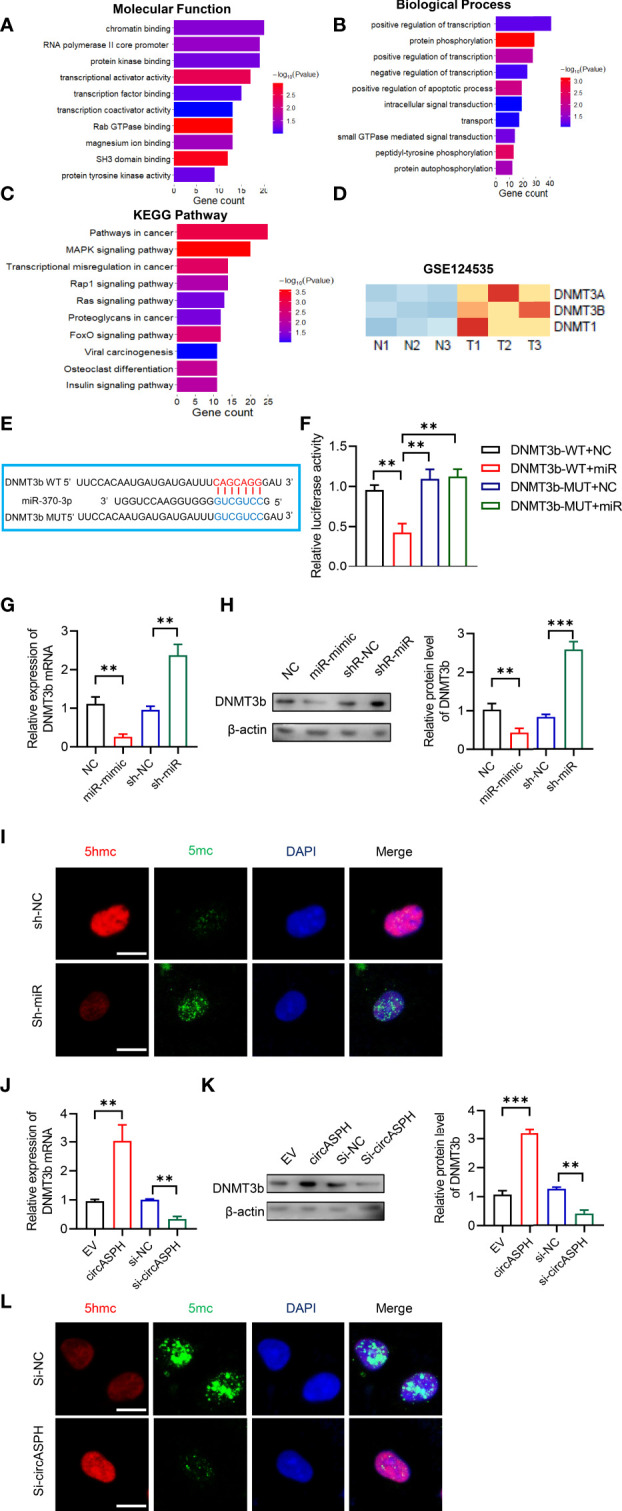
CircASPH regulated the level of the DNMT3b/5mC Axis by sponging miR-370-3p in HCC cells. **(A, B)** Molecular function and biological process of miR-370-3p target genes in gene ontology analysis. **(C)** KEGG analysis of miR-370-3p target genes. **(D)** Heatmap of the expression of the DNMT gene family level in GSE124535. **(E)** Predicted binding sites between DNMT3b and miR-370-3p. **(F)** Dual-luciferase reporter assay showed that miR-370-3p could interact with the 3’UTR of DNMT3b mRNA. **(G, H)** The expression of the DNMT3b level with miR-370-3p overexpression and knockdown was detected by RT-qPCR and Western blot. **(I)** Images of 5hmC-, 5mC-, and DAPI-stained HCC cells with different levels of miR-370-3p; scale bar = 20 μm. **(J, K)** The expression of DNMT3b level with circASPH overexpression and knockdown was detected by RT-qPCR and Western blot. **(L)** Images of 5hmC-, 5mC-, and DAPI-stained HCC cells with different levels of circASPH; scale bar = 20 μm. **p < 0.01, ***p < 0.001.

To further elucidate the molecular mechanism in which circASPH promoted HCC progression, we attempted to identify the target genes of miR-370-3p. We used the bioinformatic analysis to indicate the function of the target gene. It showed that the molecular function was enriched in chromatin binding (**Figure 4A**). In addition, the biological process was enriched in the regulation of transcription, especially in the apoptotic process (**Figure 4B**). The KEGG pathway showed that target genes mainly functioned in pathways in cancer and transcription misregulation in cancer (**Figure 4C**). Previous studies revealed that DNMT3b played a key role in establishing *de novo* DNA methylation and was upregulated in cancers. So, we used GEO databases (GSE124535) to analyze the expression of the family of DNMT. It showed that the family of DNMT was enhanced in HCC tissues (**Figure 4D**). Based on online target prediction algorithms, we found that DNMT3b was a potential candidate target gene for miR-370-3p (**Figure 4E**). The dual luciferase assay was performed, and it indicated that miR-370-3p could interact with DNMT3b (**Figure 4F**). The overexpressed miR-370-3p could decrease the DNMT3b expression level, while the upregulation of DNMT3b was induced by miR-370-3p knockdown (**Figures 4G, H**). Then, we detected changes in 5hmC and 5mC levels to assess whether miR-370-3p could remodel the epigenetic landscape by targeting DNMT3b genes and regulating 5mC levels in the genome. As expected, miR-370-3p knockdown exhibited lower levels of 5hmC and enhanced the level of 5mC (**Figure 4I**). Next, we verified the expression of DNMT3b and level of 5mC under the circASPH changed condition. The result showed that circASPH knockdown could decrease the DNMT3b expression level and decrease the level of 5mC (**Figures 4J–L**). These data indicated that circASPH could sponge miR-370-3p and regulate the DNMT3b/5mc axis in HCC cells.

### CircASPH Promotes the HCC Process *via* the miR-370-3p/DNMT3b/5mC Axis

**Figure 5:**
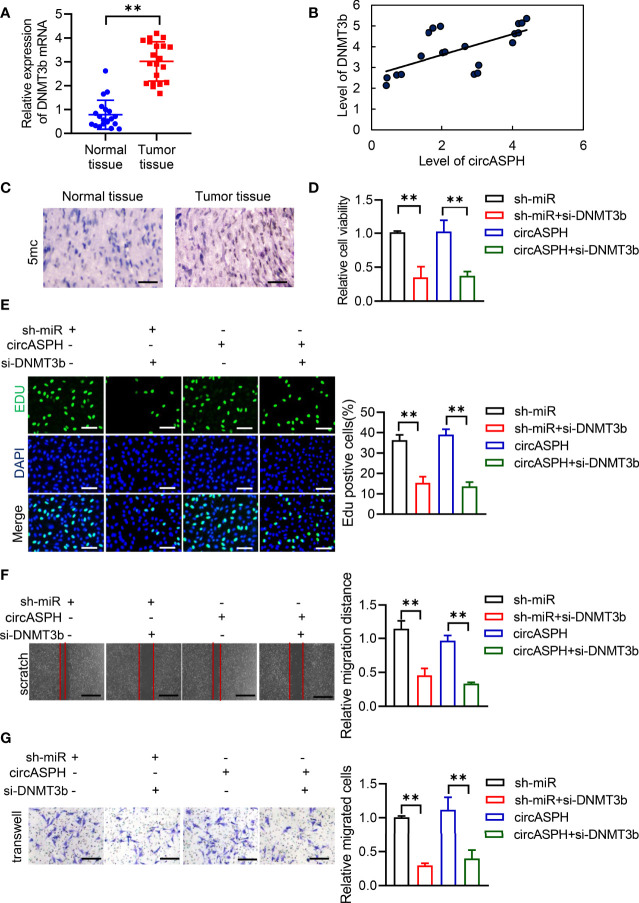
CircASPH promoted the HCC process via miR-370-3p/DNMT3b/5mC. **(A)** The level of DNMT3b expression was evaluated by RT-qPCR in 20 paired HCC tumor tissues and normal tissues. **(B)** Pearson analysis indicated the relationship between circASPH and DNMT3b. **(C)** Immunohistochemical staining for 5mC in HCC tumor tissues; scale bar = 100 μm. **(D, E)** Cell Counting Kit-8 (CCK-8) assays and EDU staining assays indicated the proliferation of cells expressing a combination of circASPH, sh-miR-370-3p, and si-DNMT3b. **(F, G)** Wound-healing assays and Transwell assays indicated the ability of migration and invasion of cells expressing a combination of circASPH, sh-miR-370-3p, and si-DNMT3b. **p < 0.01.

First, we used RT-qPCR to indicate that the level of DNMT3b was higher in HCC tumor tissues (**Figure 5A**). The Pearson analysis also showed that the expression of the DNMT3b level was positively related to the level of circASPH (**Figure 5B**). The IHC assay also showed that the 5mC level was enhanced in HCC tumor tissues (**Figure 5C**). These results prompted us to hypothesize that circASPH may promote HCC progression *via* the miR-370-3p/DNMT3b/5mC axis. DNMT3b was knocked down in HCC cell lines with circASPH overexpression or miR-370-3p knockdown, and the proliferation ability of these cells was evaluated by CCK8 assays and EDU staining assays. We found that DNMT3b knockdown significantly reduced the cell proliferation, which was enhanced by circASPH overexpression or miR-370-3p knockdown (**Figures 5D, E**). In addition, the wound-healing assays and Transwell assays also indicated that DNMT3b knockdown significantly reduced the enhanced cell migration and invasion mediated by circASPH overexpression or miR-370-3p knockdown (**Figures 5F, G**). These results suggest that the DNMT3b/5mC axis plays an important role in the function of miR-370-3p and circASPH in HCC cells.

### CircASPH Promotes HCC Progression by Regulating the DNA Methylation and Expression of HAO2

**Figure 6:**
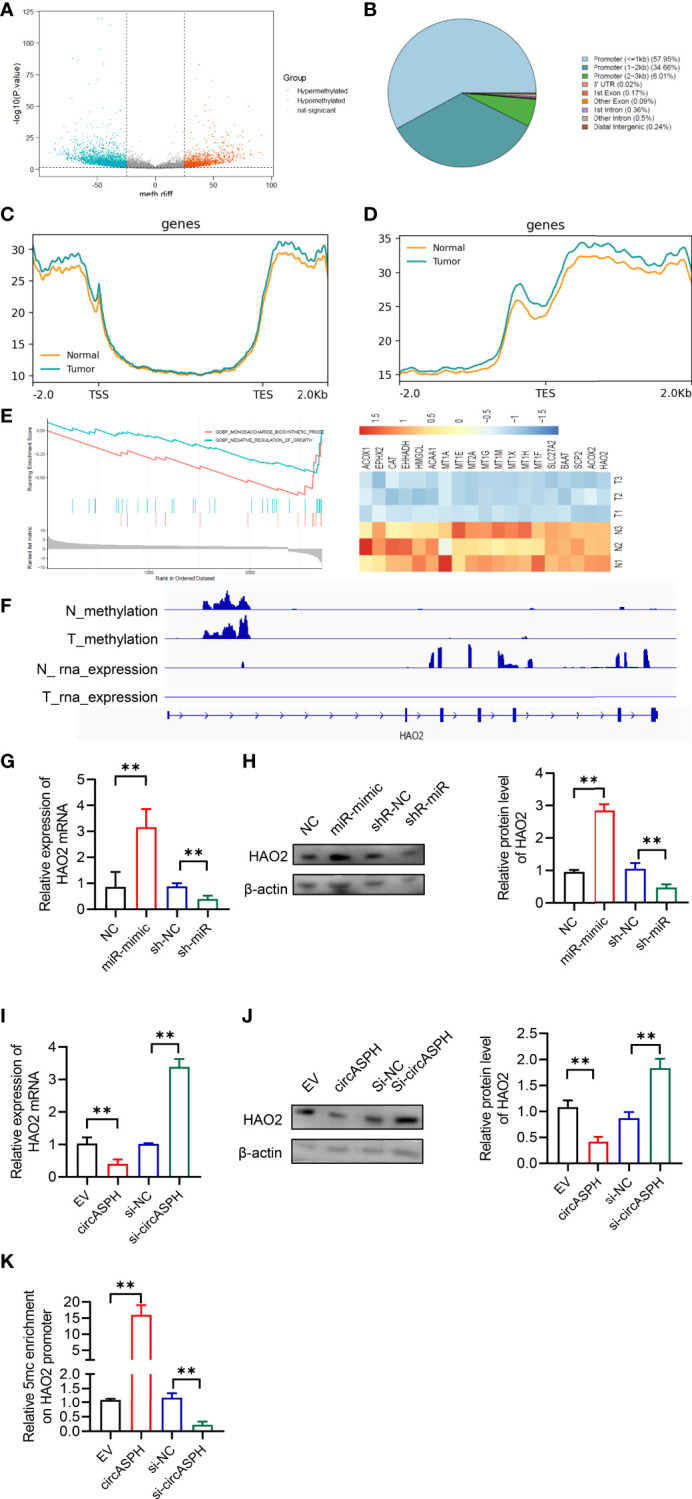
CircASPH promoted HCC progression by regulating the DNA methylation and expression of HAO2. **(A)** Volcano plot of the methylation difference between HCC tumor tissues and normal tissues. **(B)** CpG island distribution of promoter differential methylated CpG sites. **(C, D)** Average profiles of 5mC and input DNA coverage across the binding-site motif identified on 5mCþ enhancers. **(E)** GSEA analysis of the gene enriched in the negative regulation of the growth pathway. **(F)** IGV profile of 5mC-enriched regions and RNA-seq profiles in HCC tumor tissues. **(G, H)** The expression of the HAO2 level with miR-370-3p overexpression and knockdown was detected by RT-qPCR and Western blot. **(I, J)** The expression of the HAO2 level with circASPH overexpression and knockdown was detected by RT-qPCR and Western blot. **(K)** MeDIP-qPCR assays showed 5mC in HAO2 genes with different circASPH levels. **p < 0.01.

DNMT3b enzymes catalyzed 5hmC to 5mC, leading to DNA methylation and gene expression regulation. Then, we used the methylation GEO database (GSE55752) in the HCC tumor. It indicated that the DNA methylation was enhanced in HCC tumors and was enriched in the promoter area (**Figures 6A–D**). GSEA also showed that methylated genes also take part in the negative regulation of growth. In addition, HAO2 was one of the highly methylated genes in HCC tumors (**Figure 6E**). CHIP seq also showed that the methylation of HAO2 occurred in the promoter region (**Figure 6F**). Then, we examined the effect of circASPH and miR-370-3p on the HAO2 expression. Overexpressed miR-370-3p could increase the HAO2 expression level (**Figures 6G, H**). However, overexpressed circASPH could decrease the HAO2 expression level, while the upregulation of HAO2 was induced by circASPH knockdown (**Figures 6I, J**). Meanwhile, we also verified the 5mC enrichment in the HAO2 promoter region (**Figure 6K**). It indicated that circASPH overexpression could enhance the 5mC enrichment on the HAO2 promoter region and regulated the expression of HAO2.

**Figure 7:**
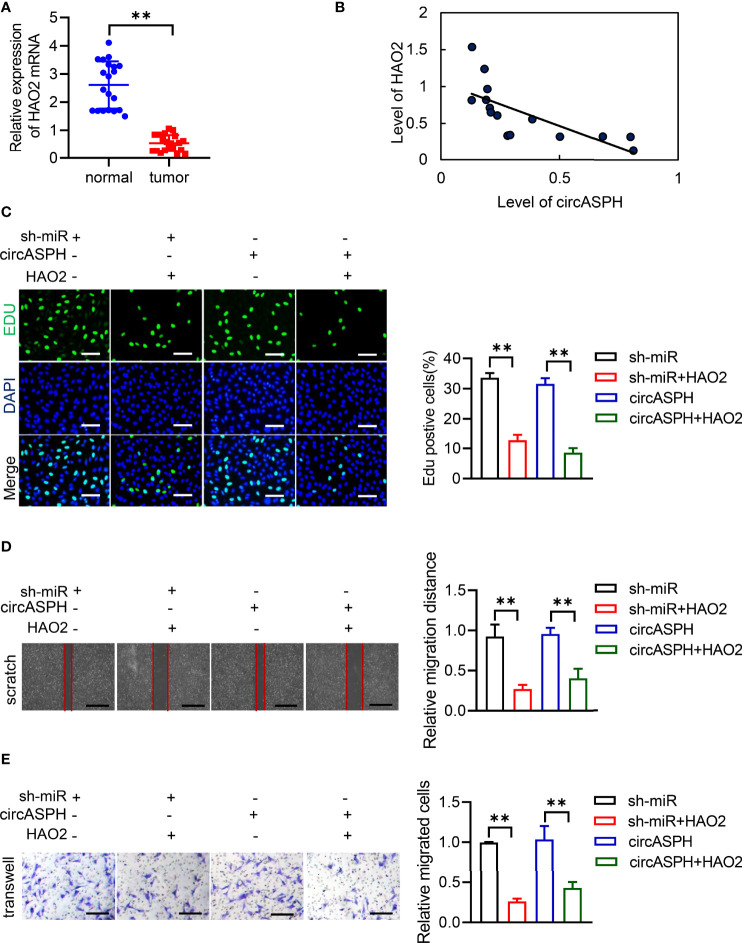
CircASPH promoted the HCC process *via* the miR-370-3p/DNMT3b/5mC/HAO2 axis. **(A)** The level of HAO2 expression was evaluated by RT-qPCR in 20 paired HCC tumor tissues and normal tissues. **(B)** Pearson analysis indicated the relationship between circASPH and HAO2. **(C)** EDU staining assays indicated the proliferation of cells expressing a combination of circASPH, sh-miR-370-3p, and HAO2. **(D, E)** Wound-healing assays and Transwell assays indicated the ability of migration and invasion of cells by expressing a combination of circASPH, sh-miR-370-3p, and HAO2. **p < 0.01.

Then, we detected the expression of HAO2 in the HCC tumor. RT-qPCR indicated that the level of HAO2 was decreased in HCC tumor tissues (**Figure 7A**). The Pearson analysis also showed that the expression of the HAO2 level was negatively related to the level of circASPH (**Figure 7B**). HAO2 was overexpressed in HCC cell lines with circASPH overexpression or miR-370-3p knockdown, and the proliferation ability of these cells was evaluated by EDU staining assays. We found that HAO2 overexpression significantly reduced cell proliferation, which was enhanced by circASPH overexpression or miR-370-3p knockdown (**Figure 7C**). In addition, the wound-healing assays and Transwell assays also indicated that HAO2 overexpression significantly reduced the increased cell migration and invasion mediated by circASPH overexpression or miR-370-3p knockdown (**Figures 7D, E**). These results demonstrated that circASPH promoted HCC progression by regulating the DNA promoter methylation and expression of HAO2.

## Discussion

Here, we indicated that circASPH could regulate the promoter methylation and expression of HAO2 to promote HCC progression *via* the miR-370-3p/DNMT3b/5mC axis. First, two databases were used to identify that circASPH was the only upregulated circRNA in tumor tissues in HCC patients and that the expression of circASPH level was closely related to the OS of HCC patients. The overexpression of circASPH could increase cell proliferation, migration, and invasion in HCC cells. It showed that circASPH could regulate the methylation process of the HAO2 promoter, by acting as a sponge for miR-370-3p, and miR-370-3p targeted DNMT3b and increased the 5mC level. Therefore, circASPH could promote the methylation process and regulate the expression of HAO2 in the HCC process.

CircRNAs are verified to play an important role in many diseases, especially in different types of cancer. CircASPH has been reported in other diseases. For instance, upregulated circASPH contributed to glioma cell proliferation and aggressiveness by the miR-599/AR/SOCS2-AS1 signaling pathway (22). CircASPH also promoted KGN cell proliferation through the miR-375/MAP2K6 axis in polycystic ovary syndrome (23). In lung adenocarcinoma, circASPH could be regulated by HMGA2 to promote tumor growth (24). However, the function of circASPH in HCC is still uncovered. Here, we indicated that circASPH led to increased cell proliferation, migration, and invasion in HCC cells, which could be a diagnostic and treatment biomarker for HCC treatment. The other two circRNAs that were identified in databases were circLIFR and circSOX5. It showed that an enforced expression of circLIFR enhanced HUASMC proliferation and impeded apoptosis (25). CircSOX5 promotes proliferation and inhibits the apoptosis of the HCC (26). Hence, we chose circASPH for the investigation.

DNA methylation is definite by the addition of a methyl group to the 5′ position of a CpG dinucleotide cytosine pyrimidine ring. Active demethylation could be regulated by the ten–eleven translocation (TET) enzyme family, including Tet 1, Tet 2, and Tet 3. 5mC and 5hmC could also also changed to thymine or 5-hydroxymethyluracil (5-hmU), depending on an alternative pathway (27). In HCC, TET1 and TET2 have been reported to be downregulated in tumor tissues and regulate DNA methylation (28–30). The DNMT1-mediated methylation of BEX1 regulates stemness and tumorigenicity in liver cancer (31). However, the mechanism of DNMT3b/5mC activation and whether the DNMT3b/5mC axis is involved in the regulation of HCC remain unresolved. Here, we identified circASPH as an important noncoding RNA that regulated the expression of DNMT3b and the 5mC level in HCC progression. Mechanistically, circASPH acted as the sponge of miR-370-3p, and miR-370-3p could bind to the 3′-UTR of DNMT3b mRNAs in HCC cells and upregulate 5mC levels. In HCC samples, there was a positive correlation between circASPH and DNMT3b expression. These data supported the conclusion that circASPH could regulate the level of the DNMT3B/5mC axis and act as a key epigenetic modifier in HCC. There are also multiple targets that can be methylated. According to GEO databases, it also showed that SIRT5 downregulation is associated with increased succinylation and activity of ACOX1 and oxidative DNA damage response in HCC (32). We will investigate the function of other genes in a future study.

In conclusion, we identified that circASPH could act like an oncogene in HCC to promote cell proliferation, migration, and invasion *via* the miR-370-3p/DNMT3b/5mC/HAO2 axis. CircASPH could be considered an important epigenetic regulator in HCC progression.

## Data Availability Statement

The datasets presented in this study can be found in online repositories. The names of the repository/repositories and accession number(s) can be found in the article/**Supplementary Material**.

## Ethics Statement

The animal study was reviewed and approved by the ethics committee of the First Affiliate Hospital of Nanjing Medical University Ethics Review Board.

## Author Contributions

CW and ZT designed the research. HZ and JX wrote the paper. JZ, JT, SH, QZ, and FZ performed the *in vitro* experiments. HZ, JX, ZX, and DS analyzed data. All authors contributed to the article and approved the submitted version.

## Funding

The study was supported by grants from the National Natural Science Foundation of China (Grant no. 81972675). The work was also supported in part by the Program for Development of Innovative Research Teams in the First Affiliated Hospital of NJMU, the Priority Academic Program of Jiangsu Higher Education Institutions.

## Conflict of Interest

The authors declare that the research was conducted in the absence of any commercial or financial relationships that could be construed as a potential conflict of interest.

## Publisher’s Note

All claims expressed in this article are solely those of the authors and do not necessarily represent those of their affiliated organizations, or those of the publisher, the editors and the reviewers. Any product that may be evaluated in this article, or claim that may be made by its manufacturer, is not guaranteed or endorsed by the publisher.
