# Supplementary material for: CircASPH Promotes Hepatocellular Carcinoma Progression Through Methylation and Expression of HAO2
Source: Front Oncol. 2022 Jun 20;12:911715. doi: 10.3389/fonc.2022.911715 (PMC9252593; doi:10.3389/fonc.2022.911715)

Figure1E

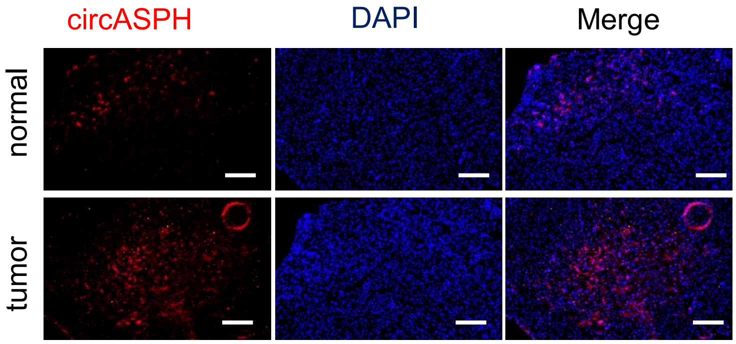

Figure2C

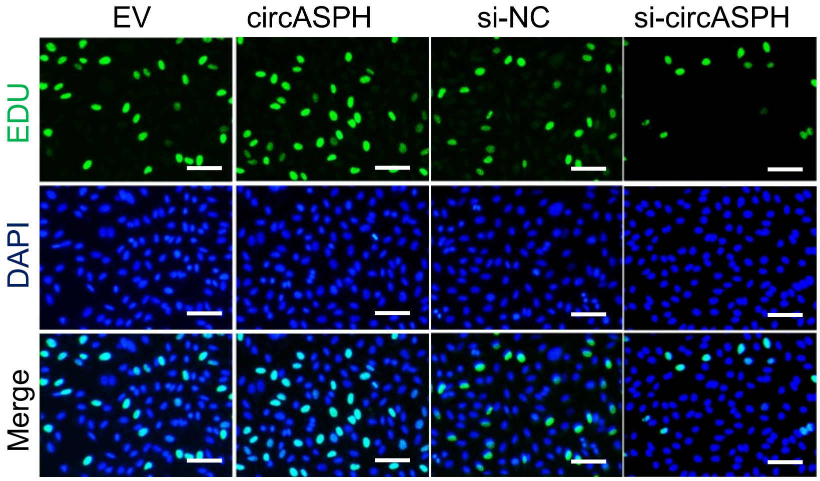

Figure2D

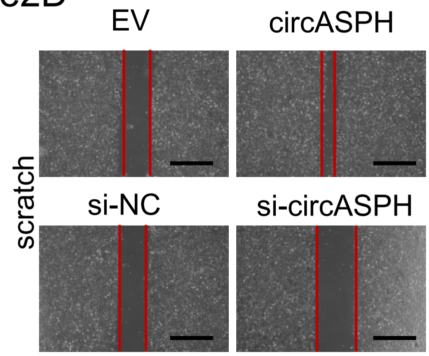

Figure2E

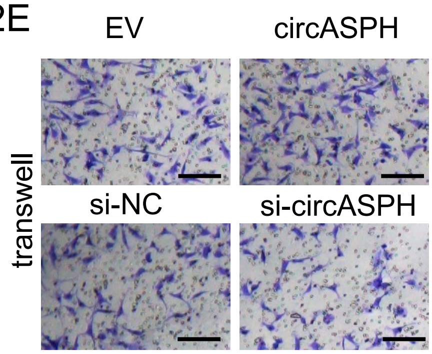

Figure2F

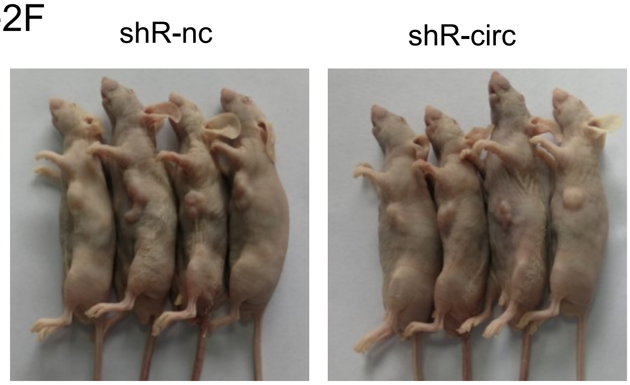

Figure3E

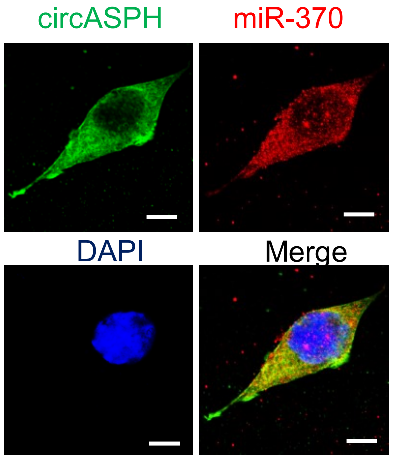

Figure4G

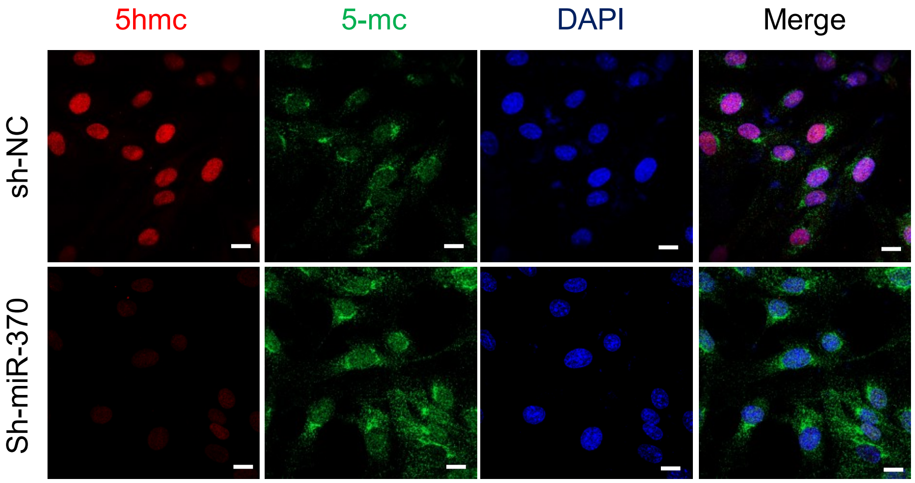

Figure4H

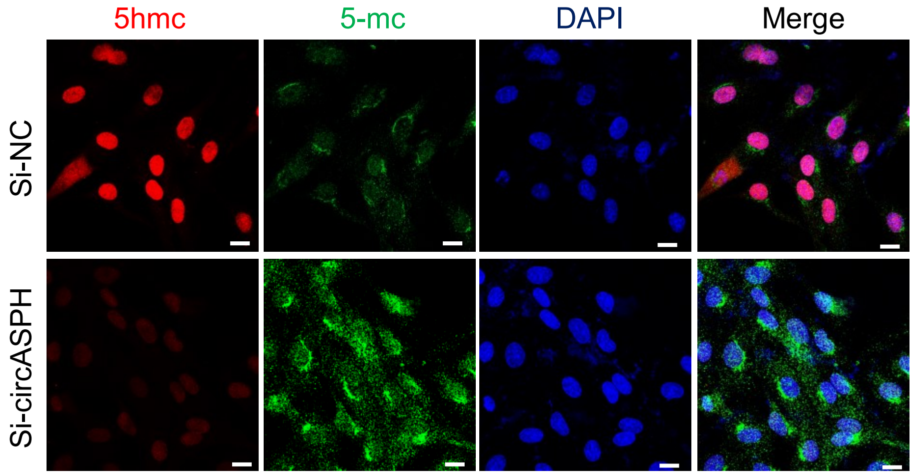

Figure5C

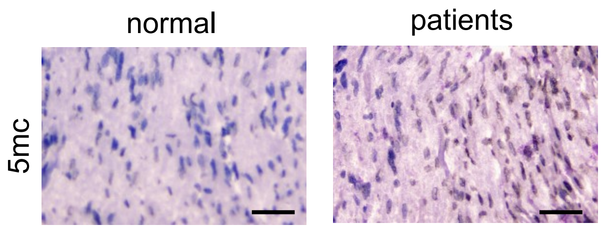

Figure5E

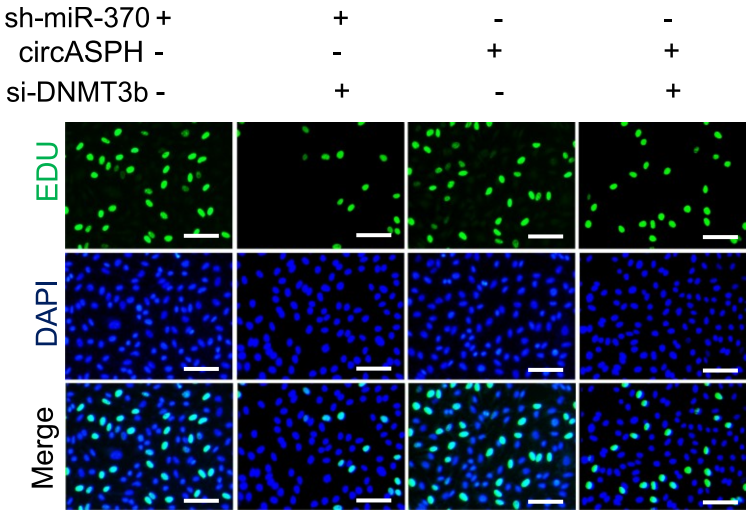

Figure5F

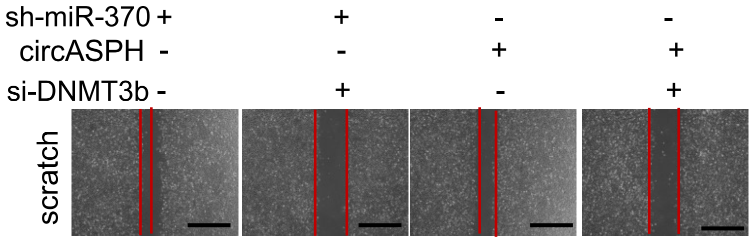

Figure5G

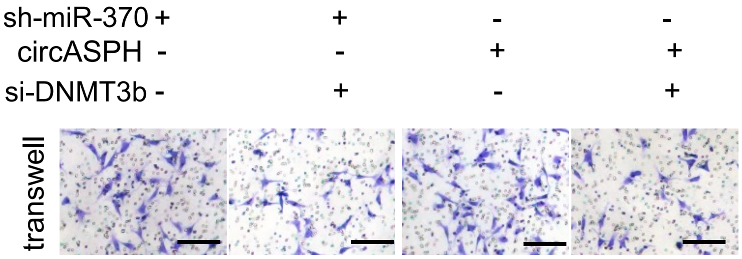

Figure6C

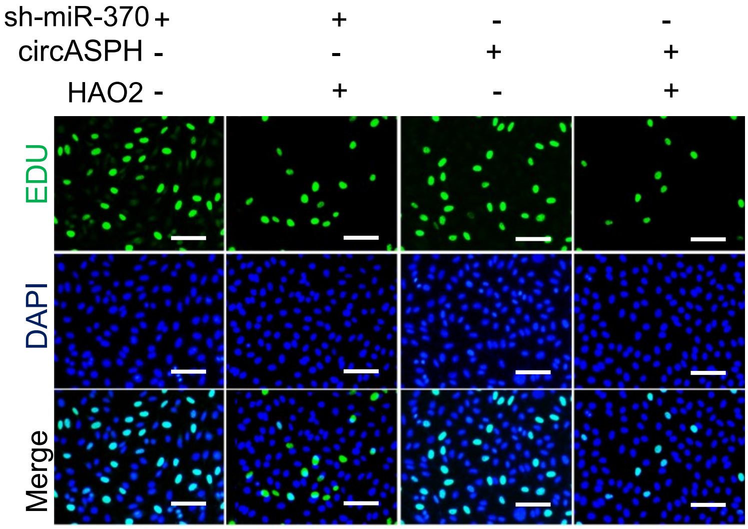

Figure6D

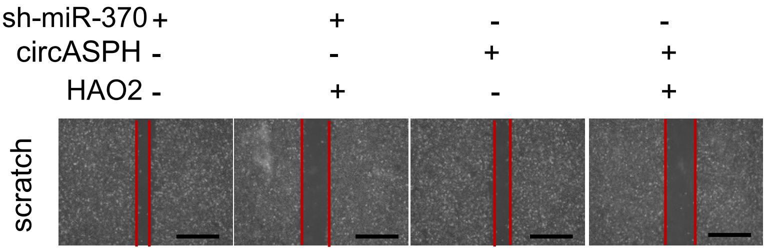

Figure6E

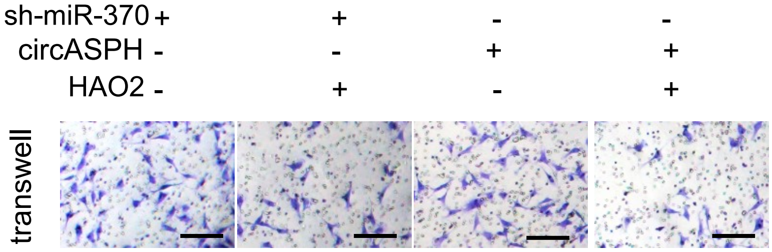

Supplement: Supplementary file 2 [file DataSheet_2.pdf]
